# A Case of Laparoscopic Mesenteric Cyst Excision

**DOI:** 10.1155/2012/594095

**Published:** 2012-09-04

**Authors:** Vikalp Jain, Jonas P. DeMuro, Matthew Geller, Elena Selbs, Carlos Romero

**Affiliations:** ^1^Department of Surgery, Stony Brook School of Medicine, Stony Brook, NY 11794, USA; ^2^Department of Surgery, Winthrop University Hospital, Mineola, NY 11501, USA; ^3^Division of Trauma & Critical Care, Department of Surgery, Winthrop University Hospital, 259 First Street, Mineola, NY 11501, USA; ^4^Department of Pathology, Winthrop University Hospital, Mineola, NY, USA

## Abstract

The objective of this study is to discuss the presentation, diagnosis, and surgical management of a young, healthy patient with a symptomatic mesenteric cyst. He had a 5-month history of abdominal pain from this disorder, and the case is presented to illustrate the clinical picture and operative management of this rare disorder.

## 1. Introduction

Mesenteric cysts are benign lesions that are found within the abdomen. They have an incidence that is less than 1 in 100,000 patients [1]. While they were first described in 1507 as an autopsy finding, [2] subsequently, less than 1000 have been described in the literature.

These lesions can present with symptoms such as abdominal pain, nausea, vomiting, anorexia, and a change in bowel habits, however, most commonly they are asymptomatic, and detected incidentally via physical exam, or imaging. Although most mesenteric cysts are benign, these lesions do occasionally cause complications, including intestinal obstruction, volvulus, torsion, or even hemorrhagic shock secondary to bleeding or rupture [3].

This case was presented at the Annual Clinic Day of the Combined Meeting of the Brooklyn & Long Island Chapters of the American College of Surgeons and the Nassau Surgical Society, December 7, 2011, General Surgery & Vascular Section.

## 2. Case Presentation

A 23-year old male presented to his primary care physician's office with a five month history of vague abdominal pain. The pain was described as intermittent and was located in the left lower quadrant. The pain was not brought on by any particular events, nor was it relieved by anything specific. The patient had six episodes of pain in the previous five months, each lasting approximately one hour.

**Figure 1:**
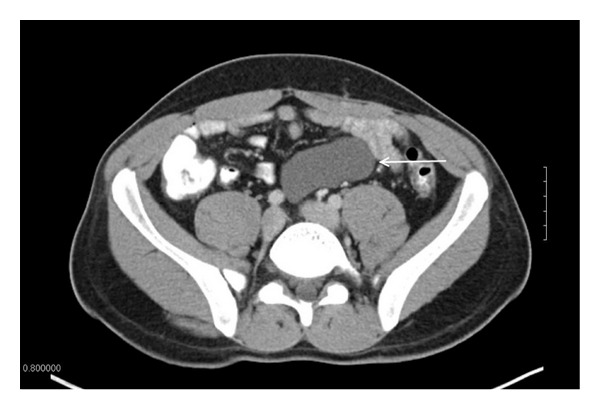
CT of the abdomen and pelvis shows an 8 cm homogenous cystic lesion (white arrow).

The patient had a past medical and surgical history that was only significant for a congenital megaureter on the left side, for which he underwent ureteral reconstruction as an infant, with no further issues. Prior to our operation for the mesenteric cyst, he was found to have normal laboratory values, including markers for malignancy. A preoperative CT of the abdomen (Figure 1) showed a 7.8 × 8.0  cm intraabdominal, homogenous cystic lesion which was unilocular, with a thin capsule located on the mesentery of the small intestine.

**Figure 2:**
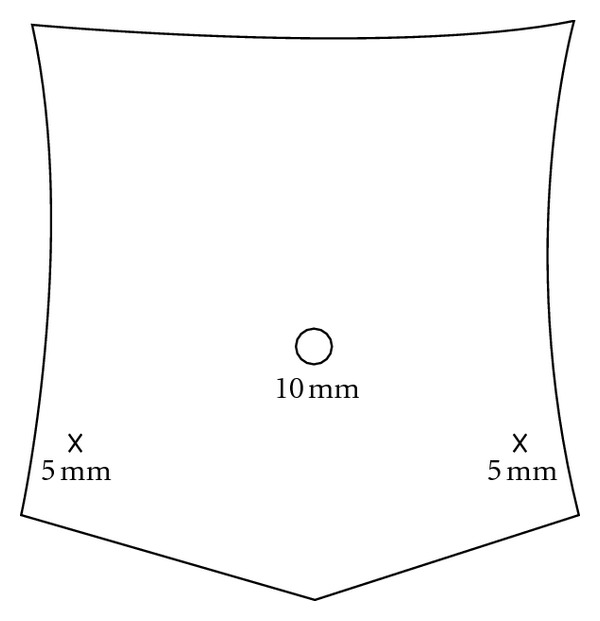
Configuration of the three ports that were utilized for the operative excision of the cyst.

Based on the preoperative CT, a minimally invasive operation was chosen. A total of three trocars were used for the surgery. Initially, a 10 mm trocar was placed at the umbilicus via the open Hasson technique, with two additional bladeless 5 mm trocars placed under direct vision in the bilateral lower quadrants (Figure 2).

**Figure 3:**
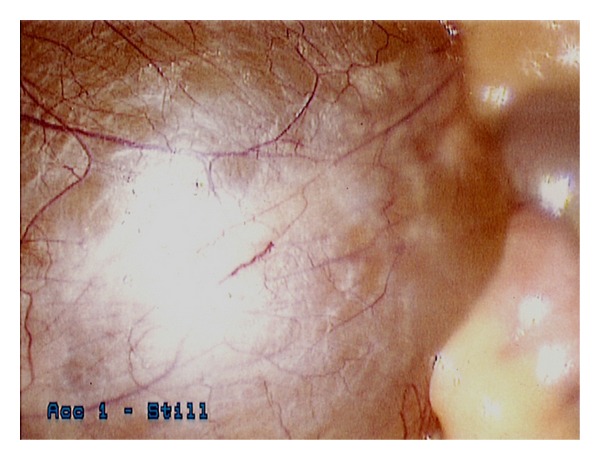
An intraoperative laparoscopic image reveals the outer capsule of the mesenteric cyst, with surface vascularity.

During the laparoscopic exploration, it was noted that the mesenteric cyst was in the left lower quadrant. It was a thin walled structure, with yellow fluid, and was fixed to the mesentery posteriorly, with no other points of attachment (Figure 3). The cyst was mobilized from the mesentery using the harmonic shears and a suction irrigator. Once free, the contents were aspirated completely, and the remaining cyst wall was removed from the abdomen via the 10 mm port with a surgical retrieval bag. The patient was discharged home the following day, tolerating a regular diet, and subsequently made a complete recovery.

**Figure 4:**
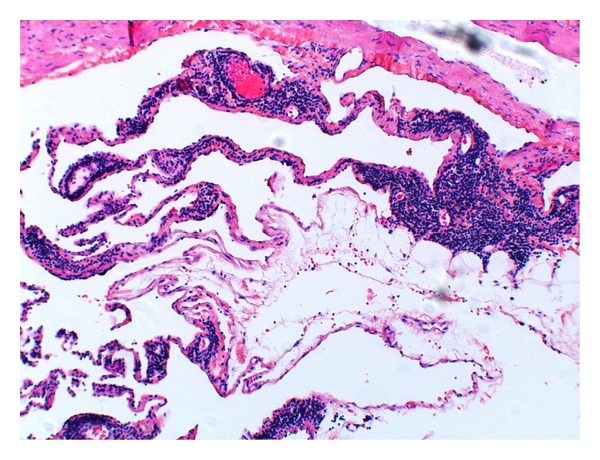
A high-power photomicrograph shows an endothelial-lined thin lymphatic, and thicker vascular channels with areas of lymphoid tissue.

Pathological examination of the cyst revealed the fluid to be benign. The wall of the cyst was found to be fibrous with a histology consistent with a lymphangioma (Figure 4).

## 3. Discussion

Mesenteric cysts are rare lesions that commonly are asymptomatic, and found incidentally. However, they can also present with abdominal pain that can be due to rupture, torsion, acute hemorrhage, or compression of nearby structures. In addition, patients can present with nonspecific symptoms, such as anorexia, nausea, vomiting, fatigue, and weight loss.

The presence of these symptoms makes it important to assure that these lesions are benign. There have been similar lesions that have been found to be cystic lymphangiomas, cystic stromal tumors, and mesotheliomas on pathologic analysis. In addition, rarely, carcinomas have been found to arise in mesenteric cysts [4]. 

The etiology of these cysts still remains unclear. It is often discussed that they are a result of degeneration of mesenteric lymphatics, or a congenital anomaly. However, they may also result from a number of etiologies, including previous pelvic surgery (as in our patient), trauma, pelvic inflammatory disease, endometriosis, or neoplasia.

The most common physical finding for a mesenteric cyst is Tillaux's sign. This is described as a mass lesion of the abdomen only mobile in the horizontal and not the vertical direction (i.e.: the patient's left to right, or right to left). Even with the presence of Tillaux's sign, it is important to confirm the diagnosis via CT, ultrasound, or MRI. In addition, preoperative imaging can help guide the decision to plan the operation laparoscopically or via an open approach [5].

The first report of a mesenteric cyst was in 1507 and was actually found during an autopsy by Benevenni in Italy, and in 1880 the first successful surgical resection of a mesenteric cyst was performed by Tillaux. Due to the rarity of mesenteric cysts, it was not until 1993 that the first successful laparoscopic surgery was reported by Mackenzie et al. [6] and subsequently only a handful of laparoscopic cases have been reported [6–13].

Therapy for these cysts should only be sought if they are symptomatic or cause complications. Treatment modalities that have been attempted include simple drainage, enucleation, marsupialization, and excision. Although many modalities exist, surgical excision is considered the mainstay for therapy, as recurrence of the cyst can occur with lesser procedures. It should be noted that with cysts that are adherent, it may be necessary to remove part of the mesentery with the mass.
